# The unfolded protein response in multiple sclerosis

**DOI:** 10.3389/fnins.2015.00264

**Published:** 2015-07-29

**Authors:** Sarrabeth Stone, Wensheng Lin

**Affiliations:** ^1^Department of Neuroscience, University of MinnesotaMinneapolis, MN, USA; ^2^Institute for Translational Neuroscience, University of MinnesotaMinneapolis, MN, USA

**Keywords:** unfolded protein response, endoplasmic reticulum stress, pancreatic endoplasmic reticulum kinase, multiple sclerosis, experimental autoimmune encephalomyelitis, oligodendrocytes, demyelination, remyelination

## Abstract

The unfolded protein response (UPR) occurs in response to endoplasmic reticulum (ER) stress caused by the accumulation of unfolded or misfolded proteins in the ER. The UPR is comprised of three signaling pathways that promote cytoprotective functions to correct ER stress; however, if ER stress cannot be resolved the UPR results in apoptosis of affected cells. The UPR is an important feature of various human diseases, including multiple sclerosis (MS). Recent studies have shown several components of the UPR are upregulated in the multiple cell types in MS lesions, including oligodendrocytes, T cells, microglia/macrophages, and astrocytes. Data from animal model studies, particularly studies of experimental autoimmune encephalomyelitis (EAE) and the cuprizone model, imply an important role of the UPR activation in oligodendrocytes in the development of MS. In this review we will cover current literature on the UPR and the evidence for its role in the development of MS.

## ER stress and UPR

**Figure 1:**
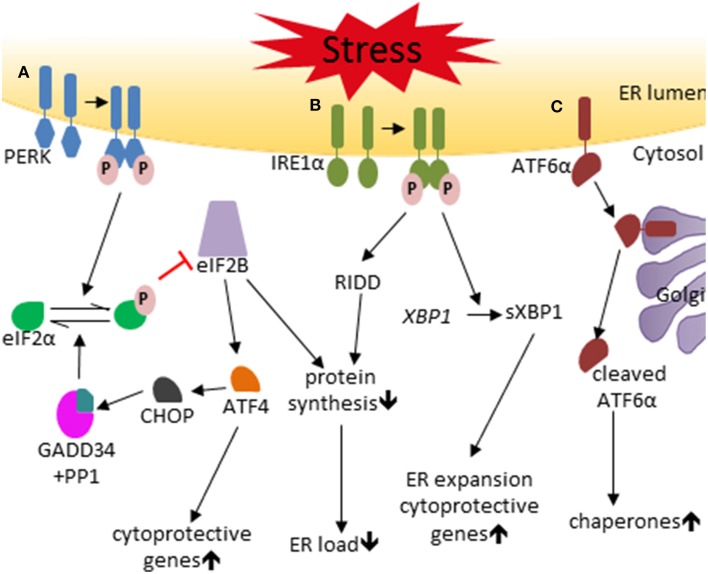
**The unfolded protein response**. **(A)** The PERK pathway. Under conditions of ER stress the PERK protein becomes activated by homodimerization and autophosphorylation. p-PERK phosphorylates eIF2α which represses eIF2B, resulting in reduced ER load through inhibition of global protein synthesis and inducation of cytoprotective genes by preferentially stimulating translation of ATF4. ATF4 also enhances the expression of CHOP, which negatively regulates p-eIF2α levels through the production of GADD34 that binds PP1 and dephosphorylates eIF2α. **(B)** The IRE1 pathway. IRE1 is activated by homodimerization and autophosphorylation in conditions of ER stress. p-IRE1 splices XBP1 mRNA to produce the sXBP1, which induces cytoprotective genes and ER expansion. IRE1 also induces mRNA degradation via RIDD to help reduce ER load. **(C)** The ATF6 pathway. ER stress results in the translocation of ATF6 to the Golgi complex where it is activated by proteolytic cleavage by the proteases S1P and S2P resulting in the 50 kDa cleaved ATF6 fragment, which stimulates the expression of chaperones.

The endoplasmic reticulum (ER) is one of the largest cellular organelles in eukaryotic cells, and consists of two types, rough ER and smooth ER (Kaufman, 1999; Csala et al., 2006; Schröder, 2008). Secretory and membrane proteins that are synthesized by ribosomes on the cytosolic surface of the rough ER are properly modified and folded inside the ER lumen. The cytoplasmic side of the smooth ER membrane is the site of biosynthesis of steroids, cholesterol, and other lipids. Additionally, the ER lumen is the major storage site for cellular calcium. Perturbations in ER homeostasis, such as elevated rates of secretory or membrane protein biosynthesis, elevated rates of lipid biosynthesis, and decreased calcium concentration, can disrupt protein modification and folding, resulting in the accumulation of unfolded or misfolded proteins in the ER lumen. This build up of unfolded or misfolded proteins is known as ER stress and results in the activation of the unfolded protein response (UPR, Figure 1) (Marciniak and Ron, 2006; Walter and Ron, 2011; Wang and Kaufman, 2012). Three ER-transmembrane proteins have been identified as the transducers of the UPR, pancreatic endoplasmic reticulum kinase (PERK), inositol-requiring enzyme 1 (IRE1), and activating transcription factor 6 (ATF6). The primary function of the UPR is to restore ER homeostasis and to adapt cells to ER-stressed condition; however, if the adaptive measures fail to restore ER homeostasis, the UPR triggers apoptosis programs to eliminate stressed cells.

The PERK protein contains an ER transmembrane domain, a cytosolic kinase domain, and a regulatory luminal domain (Harding et al., 1999, 2002). The regulatory luminal domain senses ER stress and induces PERK activation via oligomerization and autophosphorylation. Phosphorylated PERK (p-PERK) then phosphorylates the α subunit of eukaryotic translation initiation factor 2 (eIF2α). Phosphorylation of eIF2α inhibits global protein biosynthesis by suppressing the activity of eIF2B, with the two proteins forming a non-productive phosphorylated eIF2α (p-eIF2α)-eIF2B complex; the outcome of this pathway is a reduction in ER load through inhibition of nascent peptide production, preventing further ER stress (Pavitt and Proud, 2009). Despite inhibiting global protein synthesis, p-eIF2α preferentially stimulates translation of the transcription factor ATF4. Induction of ATF4 increases the expression of certain cytoprotective genes (Harding et al., 2001, 2003). In addition, ATF4 also induces CAATT enhancer binding protein homologous protein (CHOP) expression, which in turn induces growth arrest and DNA damage 34 (GADD34) expression (Marciniak et al., 2004). GADD34 functions as a regulatory subunit of a phosphatase complex that also contains protein phosphatase 1 (PP1), which specifically dephosphorylates p-eIF2α (Novoa et al., 2001). This forms a tight negative feedback loop to down-regulate the PERK-eIF2α pathway, resulting in the restoration of protein synthesis. PERK activation adapts cells to ER-stressed conditions through moderate inhibition of global protein biosynthesis and induction of cytoprotective genes. Nevertheless, PERK activation can also trigger cell apoptosis through strong inhibition of global protein biosynthesis and induction of CHOP, which function as a pro-apoptotic transcription factor (Tabas and Ron, 2011; Hetz, 2012).

Similarly to PERK, IRE1 is activated through oligomerization and autophosphorylation during ER stress. Activated IRE1 splices the mRNA of the transcription factor X-box binding protein 1 (XBP1) by its endoribonuclease activity to produce the active transcription factor spliced XBP1 (sXBP1). sXBP1 enhances the expression of chaperones and certain cytoprotective genes, and promotes ER expansion (Chen and Brandizzi, 2013; Maurel et al., 2014). Activated IRE1 also promotes the degradation of certain mRNAs to reduce the ER load through regulated IRE1-dependent decay (RIDD). Moreover, activated IRE1 can induce activation of the JUN amino-terminal kinase (JNK) and the apoptosis signal-regulating kinase 1 (ASK1) by binding to certain adaptor proteins. The third transducer of the UPR, ATF6, transits to the Golgi complex during ER stress, where it is cleaved by the proteases S1P and S2P. Cleaved ATF6 functions as a transcription factor that enhances the expression of chaperones and certain cytoprotective genes (Shen et al., 2002; Glembotski, 2014).

## Activation of the UPR in MS and EAE lesions

Multiple sclerosis (MS) is a T cell-mediated autoimmune demyelinating disease of the central nervous system (CNS) (Frohman et al., 2006; Bradl and Lassmann, 2010). The pathological hallmark of MS is the presence of multiple demyelinated plaques in the CNS white matter, which are characterized by inflammation, oligodendrocyte loss, demyelination, and axon degeneration. Although the etiology of MS remains unknown, MS is thought to be initiated by an autoimmune reaction against oligodendrocytes and myelin. It is generally believed that T cells are activated by myelin components in the peripheral immune system in MS patients, and then myelin reactive T cells cross the blood-brain barrier, entering the CNS where they initiate inflammation. CNS inflammation in MS includes infiltration of T cells, B cells, and monocytes, activation of macrophages and microglia, gliosis, upregulation of major histocompatibility complex (MHC) molecules, and elevated levels of inflammatory cytokines, and reactive oxygen and nitrogen species. This inflammatory environment leads to oligodendrocyte death, demyelination, and axon degeneration. Once inflammation decreases, oligodendrocytes can regenerate and remyelination can occur in the demyelinated lesion. However, this remyelination is insufficient to repair myelin damage, resulting in a loss of neurological function in MS patients (Franklin, 2002; Franklin and Ffrench-Constant, 2008).

Experimental autoimmune encephalomyelitis (EAE) is the primary animal model used in MS research that displays many of the clinical, pathological, and immunological features of MS (Steinman, 1999; Gold et al., 2006). Unlike MS, which is a spontaneous disease of unknown etiology, EAE is induced by immunizing animals (most commonly mice) with myelin or myelin components, such as proteolipid protein (PLP), myelin basic protein (MBP) or myelin oligodendrocyte glycoprotein (MOG) (Merrill et al., 1992; Baxter, 2007). This leads to exposure of the peripheral immune cells to myelin components and results in activation of myelin specific T cells, which then migrate into the CNS where they induce inflammatory demyelination in a manner similar to that seen in MS lesions (Mor and Cohen, 1992; Baxter, 2007). EAE has successfully been used to develop several drugs for the treatment of MS, including glatiramer acetate, and natalizumab (Baxter, 2007).

The primary function of oligodendrocytes is to produce the myelin sheath which wraps around axons, insulating and protecting them (Bankston et al., 2013; Nave and Werner, 2014). Several lines of evidence have suggested that oligodendrocyte death induced by inflammatory attacks contributes significantly to the development of MS and EAE (Prineas and Parratt, 2012; Lin et al., 2013). Oligodendrocyte apoptosis has been identified as the earliest structural change in newly forming demyelinating lesions in both MS and EAE by a number of studies (Barnett and Prineas, 2004; Lin et al., 2013). Both oligodendrocyte-specific expression of anti-apoptotic proteins (such as p35, a viral caspase inhibitor) and oligodendrocyte-specific deletion of pro-apoptotic proteins (such as tumor necrosis factor receptor 1; TNF-R1, Fas, and Fas-Associated protein with Death Domain; FADD) protect oligodendrocytes against inflammatory attacks, resulting in attenuation of EAE disease severity and amelioration of demyelination, axonal degeneration, and inflammation in EAE lesions (Hisahara et al., 2000, 2003; Hövelmeyer et al., 2005; McGuire et al., 2010). A number of reports have also shown that enhancing survival signaling pathways in oligodendrocytes protects mice against EAE, while impairing survival signaling pathways in oligodendrocytes renders mice susceptible to EAE (Balabanov et al., 2007; Ren et al., 2011; Lin et al., 2013; Hussien et al., 2014). Additionally, oligodendrocyte regeneration in demyelinated lesions is essential and necessary for remyelination and restoration of neurological functions in MS and EAE (Franklin, 2002; Franklin and Ffrench-Constant, 2008).

The UPR plays a critical role in inflammatory diseases (Zhang and Kaufman, 2008; Kitamura, 2011). Elevated levels of inflammatory mediators, such as immune cytokines, reactive oxygen species, and reactive nitrogen species, can activate the UPR in tissue resident cells and infiltrated inflammatory cells in inflammatory lesions. Activation of the UPR has been observed in multiple cell types in MS and EAE lesions (Lin and Popko, 2009). Microarray analysis revealed elevated levels of the UPR-responsive genes ATF4 and heat shock protein 70 in MS demyelinated lesions (Cwiklinska et al., 2003; Mycko et al., 2004). Real-time PCR analysis showed increased mRNA levels of the UPR makers ATF4, immunoglobulin-heavy-chain-binding protein (BiP), and CHOP in normal-appearing white matter and demyelinating lesions in the CNS of MS patients (Cunnea et al., 2011). Using biopsy specimens and post-mortem samples, immunohistochemistry analysis demonstrated increased expression of the UPR markers CHOP and BiP in oligodendrocytes, astrocytes, T cells and macrophages/microglia in MS lesions (Mháille et al., 2008; Cunnea et al., 2011; McMahon et al., 2012; Ní Fhlathartaigh et al., 2013). Moreover, elevated levels of several UPR markers, including p-PERK, p-eIF2α, BiP, and CHOP, have been demonstrated in oligodendrocytes, T cells, astrocytes, and macrophages/microglia during the course of EAE (Chakrabarty et al., 2004, 2005; Lin et al., 2007; Deslauriers et al., 2011; Ní Fhlathartaigh et al., 2013; Meares et al., 2014).

## The dual roles of IFN-γ in models of MS are mediated by the UPR

It has been well-documented that the T cell-derived pleiotropic cytokine interferon-γ (IFN-γ) plays a critical role in the development of MS and EAE (Popko et al., 1997; Lees and Cross, 2007; Goverman, 2009). A large number of studies have shown that IFN-γ promotes myelin damage and oligodendrocyte death in immune-mediated demyelinating diseases by stimulating inflammation, including activation of macrophages/microglia, upregulation of MHC molecules, and induction of inflammatory mediators (reviewed in Goverman, 2009 as well as Popko and Baerwald, 1999). Indeed, treatment of MS patients with IFN-γ results in exacerbation of symptoms (Panitch et al., 1987). Nevertheless, another set of reports showed that IFN-γ has protective effects during EAE (Mühl and Pfeilschifter, 2003; Wheeler and Owens, 2005). Both IFN-γ deficient mice and IFN-γ receptor (IFN-γR) deficient mice develop a more severe EAE disease course compared to wild type control mice (Ferber et al., 1996; Willenborg et al., 1996). Using transgenic mice that allow for temporally regulated expression of IFN-γ in the CNS using a tetracycline controllable system (Lin et al., 2004), studies have shown that the beneficial or detrimental effects of IFN-γ on the development of EAE are dependent on the timing of its presence in the CNS (Lin et al., 2006, 2007). CNS-expression of IFN-γ before EAE onset ameliorates the disease course and prevents EAE-induced oligodendrocyte loss, demyelination, and axon degeneration (Lin et al., 2007). In contrast, CNS-expression of IFN-γ at the recovery stage of EAE impairs the disease recovery and suppresses oligodendrocyte regeneration and remyelination in demyelinated lesions (Lin et al., 2006). Importantly, these studies also demonstrated that both the beneficial and detrimental effects of IFN-γ on the development of EAE are mediated, at least in part, by activation of the PERK-eIF2α pathway in oligodendrocytes (Lin et al., 2006, 2007).

Lin et al. (2007) reported that CNS-expression of IFN-γ before EAE onset almost completely blocked oligodendrocyte death, demyelination, and axonal degeneration in the CNS of EAE mice. Modest activation of the PERK-eIF2α pathway is detected in the oligodendrocytes of control EAE mice; CNS-expression of IFN-γ before EAE onset markedly enhances activation of the PERK-eIF2α pathway in oligodendrocytes (Lin et al., 2007). Importantly, CNS delivery of IFN-γ before EAE onset does not ameliorate the severity of disease course or prevent EAE-induced oligodendrocyte loss, demyelination, and axon degeneration in mice on the PERK heterozygous deficient background. In fact, MBP immunohistochemistry and toluidine blue staining show there is more severe myelin damage in the CNS of PERK heterozygous deficient mice that express transgenic IFN-γ in the CNS before EAE onset than the control EAE mice (Lin et al., 2007). However, PERK heterozygous deficiency alone does not alter EAE disease course, tissue damage in the CNS, or the immune responses in the peripheral immune system or CNS. Thus, these results demonstrate that the beneficial effect of IFN-γ in EAE is dependent on PERK signaling (Lin et al., 2007).

It is generally believed that remyelination in demyelinated lesions in adult animals largely recapitulates developmental myelination (Fancy et al., 2011). Several reports have shown that CNS-expression of IFN-γ during development causes myelinating oligodendrocyte death, hypomyelination, and inflammation (Corbin et al., 1996; LaFerla et al., 2000; Lin et al., 2008; Lin and Lin, 2010). Interestingly, a paper showed that the presence of IFN-γ in the CNS activates the PERK-eIF2α pathway in myelinating oligodendrocytes and that PERK heterozygous deficiency exacerbates IFN-γ-induced myelinating oligodendrocyte death and hypomyelination in young, developing mice (Lin et al., 2005). Moreover, another report demonstrated that GADD34 inactivation elevates the level of p-eIF2α in myelinating oligodendrocytes and attenuates myelinating oligodendrocyte death and hypomyelination in young, developing mice that express IFN-γ in the CNS (Lin et al., 2008).

Feeding of cuprizone (bis-cyclohexanone oxaldihydrazone) to young adult mice induces a synchronous consistent demyelination (Matsushima and Morell, 2001; Stidworthy et al., 2003). Oligodendrocytes undergo apoptosis in response to cuprizone treatment, followed by almost complete demyelination of the corpus callosum. After removal of cuprizone, oligodendrocytes regenerate and remyelination takes place over the course of a few weeks. The cuprizone model is considered to be one of the best mouse models to study the mechanisms of remyelination in MS (Denic et al., 2011; van der Star et al., 2012). The presence of IFN-γ in the CNS suppresses oligodendrocyte regeneration and remyelination in cuprizone-induced demyelinated lesions (Lin et al., 2006). Importantly, the presence of IFN-γ in the CNS activates the PERK-eIF2α pathway in remyelinating oligodendrocytes and PERK heterozygous deficiency exacerbates IFN-γ-induced apoptosis of remyelinating oligodendrocytes and remyelination failure in cuprizone-induced demyelinated lesions (Lin et al., 2006). Taken together, these results demonstrate that activation of the PERK-eIF2α pathway protects (re)myelinating oligodendrocytes against the detrimental effects of IFN-γ in immune-mediated demyelinating diseases.

The mechanisms by which IFN-γ induces ER stress and activates the UPR in oligodendrocytes in immune-mediated demyelinating diseases remain unknown. IFN-γ exerts its functions by binding to its receptors, IFN-γR1 and IFN-γR2 (Ramana et al., 2002). Binding of IFN-γ leads to oligomerization of its receptors and activation of the Janus kinases (JAK)1 and JAK2, resulting in trans-phosphorylation of the JAKs and the intracellular domain of the receptors, allowing interaction with STAT1. STAT1 is activated via phosphorylation of tyrosine 701, and then translocates to the nucleus where it stimulates the expression of IFN-γ-responsive genes. *In vitro* studies show that IFN-γ can induce ER stress and activate the UPR in multiple cell types, including oligodendrocytes (Lin et al., 2005; Gade et al., 2012). Using STAT1 knockout mice, a study reported that STAT1 deletion diminishes the ability of IFN-γ to activate the UPR in oligodendrocytes in the CNS (Lin and Lin, 2010). However, this study also shows that STAT1 deletion diminishes the ability of IFN-γ to induce inflammation in the CNS. Many inflammatory mediators induced by IFN-γ, such as tumor necrosis factor-α, nitrogen oxide, and hydrogen peroxide, are capable of activating the UPR in cells (Hu et al., 2006; Cao and Kaufman, 2014). Therefore, it is likely that the presence of IFN-γ in the CNS activates the UPR in oligodendrocytes in immune-mediated demyelinating diseases through both its direct actions on the cells and through induction of inflammation.

## The protective effects of moderate perk activation on oligodendrocytes in models of MS

To further dissect the precise role of PERK signaling in oligodendrocytes during immune-mediated demyelinated diseases, a mouse model (*PLP/Fv2E-PERK* mice) that allows for temporally controlled activation of PERK signaling specifically in oligodendrocytes has been generated (Lin et al., 2013). This mouse model expresses Fv2E-PERK, an artificial PERK derivative, under the control of the proteolipid protein (PLP) promoter, thus restricting its expression to oligodendrocytes in the CNS (Lin et al., 2013). Fv2E-PERK contains the effector domain of PERK (eIF2α-kinase domain) fused to two modified FK506 binding domains (Fv2E) (Lu et al., 2004). Treatment with the ligand for the Fv2E domain (AP20187) results in dimerization and autophosphorylation of the eIF2α-kinase domain allowing activation of PERK signaling to be controlled and isolated from ER stress. Importantly, AP20187 treatment activates PERK signaling in oligodendrocytes of *PLP/Fv2E-PERK* mice in a dose dependent manner. ER stress and activation of the UPR are believed to be moderate under physiological and pathological conditions (Qi et al., 2011). To mimic the moderate PERK activation under normal and disease conditions, heterozygous *PLP/Fv2E-PERK* mice are treated with a low dose of AP20187 to induce moderate activation of PERK signaling specifically in oligodendrocytes. Moderate PERK activation has no effect on mature oligodendrocytes in fully myelinated adult mice (Lin et al., 2013). Moreover, moderate PERK activation does not affect the viability or function of myelinating oligodendrocytes in young, developing mice or remyelinating oligodendrocytes in cuprizone-induced demyelinated lesions (Lin et al., 2014a).

Using this unique mouse model, a study demonstrated that moderate PERK activation in mature oligodendrocytes is cytoprotective and protects mice against EAE (Lin et al., 2013). Treating heterozygous *PLP/Fv2E-PERK* mice with a low dose of AP20187 starting before EAE onset, at post-immunization day (PID) 10, elevates the level of p-eIF2α exclusively in oligodendrocytes, significantly ameliorates the severity of EAE clinical symptoms, and attenuates EAE-induced oligodendrocytes loss, demyelination, and axon degeneration in the CNS. Oligodendrocyte loss is one of the first signs of pathological changes in EAE and can occur before demyelinating lesions are identifiable. Importantly, the low dose of AP20187 treatment significantly reduces the number of apoptotic oligodendrocytes in the CNS of heterozygous *PLP/Fv2E-PERK* mice before the onset of clinical disease and the formation of demyelinating lesions (at PID12). Moreover, moderate PERK activation in mature oligodendrocytes does not significantly affect the immune responses in CNS or peripheral immune system during EAE (Lin et al., 2013).

Similarly, using the same mouse model, another study demonstrated that moderate PERK activation promotes remyelinating oligodendrocyte survival and remyelination in demyelinated lesions in models of MS (Lin et al., 2014a). It has been shown that the presence of IFN-γ in the CNS stimulates inflammation and is detrimental to myelinating oligodendrocytes in young, developing mice and to remyelinating oligodendrocytes in demyelinated lesions in the cuprizone models (Lin et al., 2005, 2006, 2008). This study showed that moderate PERK activation specifically in myelinating oligodendrocytes protects both oligodendrocytes and myelin against the detrimental effects of IFN-γ in young, developing mice, without altering the inflammatory response induced by the cytokine (Lin et al., 2014a). Moderate PERK activation specifically in oligodendrocytes also attenuates IFN-γ-induced remyelinating oligodendrocyte apoptosis and remyelination failure in cuprizone-induced demyelinated lesions. Moreover, moderate PERK activation specifically in oligodendrocytes during the recovery stage of EAE does not change the severity of clinical symptoms, but significantly enhances oligodendrocyte regeneration and remyelination in EAE demyelination lesions. Additionally, moderate PERK activation specifically in oligodendrocytes has no effect on the inflammatory response in both cuprizone and EAE demyelination lesions (Lin et al., 2014a).

In parallel to the above findings, Hussien et al. (2014) showed that oligodendrocyte-specific PERK deletion increases the susceptibility of mice to EAE. Mice with oligodendrocyte-specific PERK deletion appear healthy and exhibit normal myelination in the CNS under physiological conditions. However, these mice develop a significantly more severe EAE disease course as compared to the control mice. Moreover, oligodendrocyte-specific PERK deletion exacerbates EAE-induced oligodendrocyte loss, demyelination and axon degeneration in the CNS. Additionally, there is no evidence that oligodendrocyte-specific PERK deletion alters the immune response in EAE mice.

Data from these cell-specific conditional mouse models that allow for manipulation of the activity of PERK signaling specifically in oligodendrocytes demonstrate that moderate PERK activation acts cell-autonomously to protect oligodendrocytes (both mature and remyelinating oligodendrocytes) against inflammatory attacks in immune-mediated demyelinating diseases (Lin et al., 2013, 2014a; Hussien et al., 2014). Although it is generally believed that PERK activation promotes cell survival under cytotoxic conditions through global attenuation of protein translation and induction of stress-induced cytoprotective genes (Marciniak and Ron, 2006; Walter and Ron, 2011), the precise molecular mechanisms responsible for the cytoprotective effects of PERK signaling on oligodendrocytes in these diseases remain unknown. Several lines of evidence have suggested that PERK signaling activates the NF-κB pathway by repressing the translation of the NF-κB inhibitor IκBα during ER stress (Jiang et al., 2003; Deng et al., 2004). Activation of NF-κB, which is increasingly recognized as an anti-apoptotic transcription factor (Karin and Lin, 2002; Mincheva-Tasheva and Soler, 2013), has been observed in oligodendrocytes in MS and EAE lesions (Yan and Greer, 2008; McGuire et al., 2013). Interestingly, recent studies have shown that PERK activation in oligodendrocytes activates the NF-κB pathway in the cells *in vitro* and *in vivo* (Lin et al., 2012, 2013). These data raise the possibility that NF-κB activation contributes to the protective effects of PERK signaling on oligodendrocytes in immune-mediated demyelinating diseases. A mouse model that allows for controllable activation of PERK and inactivation of NF-κB specifically in oligodendrocytes would be the ideal model system to test this possibility.

## Unique features of the UPR in oligodendrocytes

The myelin sheath is an enormous lipid-rich membrane structure. Each myelinating oligodendrocytes must synthesize vast amounts of myelin membrane proteins and membrane lipids through the ER to assemble the myelin sheath (Pfeiffer et al., 1993; Anitei and Pfeiffer, 2006). Not surprising, evidence suggest that oligodendrocytes are highly sensitive to disruptions in ER homeostasis (Lin and Popko, 2009). Recent studies showed that ER stress and the UPR play a critical role in a number of myelin disorders in the CNS, including MS (as described above), Pelizaeus-Merzbacher disease, vanishing white matter disease (VWMD), and spinal cord injury (D'Antonio et al., 2009; Lin and Popko, 2009; Ohri et al., 2013). Interestingly, the UPR in oligodendrocytes has its unique features.

Data from VWMD, a disease caused by mutations in the genes encoding the five subunits of eIF2B, suggest that oligodendrocytes are hyper-sensitive to activation of the PERK-eIF2α-eIF2B pathway (Scheper et al., 2006; Lin, 2015). VWMD patients exhibit profound myelin loss in the CNS white matter; however, the gray matter and other organs are generally spared. eIF2B acts as a guanine nucleotide exchange factor and participates in each translation initiation event in eukaryotic cells. The activity of eIF2B is tightly regulated by the level of p-eIF2α. A large number of studies have shown that eIF2B mutations in VWMD patients reduce its activity (reviewed in Pavitt and Proud, 2009). Interestingly, a very recent study demonstrated that impaired eIF2B activity specifically in oligodendrocytes during the active phase of myelination reproduces the characteristic features of VWMD in mice, including hypomyelinating phenotypes, premature death, foamy oligodendrocytes, and myelin loss (Lin et al., 2014b). Taken together, these data indicate that impairment of eIF2B induced by VWMD mutations predominately affects oligodendrocytes and subsequently results in the selective white matter pathology in most cases of VWMD (Lin, 2015).

Of the three key transducers of the UPR the role of PERK signaling in oligodendrocytes has been the best characterized. Additionally, there is evidence (such as induction of BiP) suggesting activation of the ATF6 branch of the UPR in oligodendrocytes (Lin et al., 2005, 2013; Mháille et al., 2008; Ní Fhlathartaigh et al., 2013). However, although the IRE1-XBP1 branch is the most conserved part of the UPR, there is no evidence that this pathway plays a major role in oligodendrocytes. Despite considerable efforts, we and other groups have never found activation of the IRE1-XBP1 pathway in oligodendrocytes *in vitro* or *in vivo* under physiological or pathological conditions. It is known that activation of PERK signaling and ATF6 signaling is notoriously difficult to detect either *in vitro* or *in vivo*. In contrast, PCR-based assays that examine XBP1 splicing are highly sensitive methods for detection of activation of the IRE1-XBP1 pathway (Cox et al., 2011). Failure to detect XBP1 splicing in oligodendrocytes suggests a minimal role for the IRE1-XBP1 pathway in oligodendrocytes.

A large number of studies have demonstrated that CHOP, an effector of the PERK-eIF2α pathway, functions as a pro-apoptosis transcription factor in many cell types during ER stress (Tabas and Ron, 2011; Hetz, 2012). In contrast, evidence suggests that CHOP induction is not detrimental to oligodendrocytes (Gow and Wrabetz, 2009). Southwood et al. (2002) showed that UPR markers such as CHOP, BiP, and ATF3 are upregulated in oligodendrocytes of *PLP1* mutant mice and that CHOP deletion exacerbates the clinical symptom and oligodendrocyte apoptosis in *PLP1* mutant mice. It has also been shown that upregulation of CHOP induced by moderate PERK activation in oligodendrocytes of heterozygous *PLP/Fv2E-PERK* mice does not have a detrimental effect on the cells under normal or disease conditions (Lin et al., 2013, 2014a). Moreover, a recent study showed that treating homozygous *PLP/Fv2E-PERK* mice with a high dose of AP20187 strongly activates PERK signaling specifically in oligodendrocytes, resulting in strong inhibition of protein biosynthesis and upregulation of CHOP in the cells. Notably, strong PERK activation inhibits the myelinating function of oligodendrocytes in young developing mice by suppressing protein translation, but does not affect oligodendrocyte viability. Intriguingly, strong PERK activation in mature oligodendrocytes of fully-myelinated adult mice had minimal effects on the function and viability of the cells (Lin et al., 2014b). Consistent with these studies, a report showed that CHOP deletion does not alter the disease severity of EAE in mice (Deslauriers et al., 2011). Collectively, these data suggest that CHOP does not act as a pro-apoptosis transcript factor in oligodendrocytes.

## Therapeutic potential of the UPR in MS

**Figure 2:**
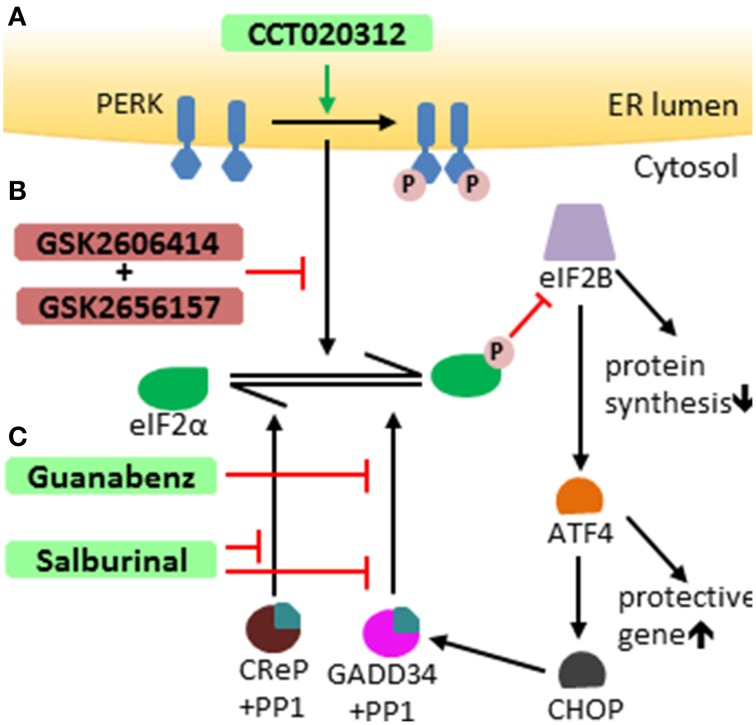
**Modulators of the PERK-eIF2 α pathway**. **(A)** CCT020312 activates PERK, inducing activation of the PERK-eIF2α pathway independently of ER stress. **(B)** GSK2606414 and GSK2656157 both selectively suppress PERK-mediated eIF2α phosphorylation. **(C)** Salburinal selectively inhibits of the activity of phosphatase complexes that dephosphorylate p-eIF2α, including both GADD34/PP1 complex and CReP (constitutive repressor of eIF2a phosphorylation)/PP1 complex. Guanabenz selectively binds to GADD34 and inhibits the activity of GADD34/PP1 complex, attenuating eIF2α dephosphorylation.

It is well-documented that the UPR influences the development of a number of human diseases, including inflammatory diseases, neurodegenerating diseases, myelin disorders, and tumors (Zhang and Kaufman, 2008; Lin and Popko, 2009; Hetz and Mollereau, 2014; Wang and Kaufman, 2014). Recent studies suggest that manipulation of the UPR pathways has therapeutic potential (Hetz et al., 2013). A number of small chemical compounds that selectively modulate the PERK-eIF2α pathway have been documented (Figure 2). PERK inhibitors GSK2606414 and GSK2656157 are high affinity ligands of the eIF2α kinase domain of the PERK protein and suppress PERK-mediated eIF2α phosphorylation by competing for ATP (Axten et al., 2012; Harding et al., 2012; Atkins et al., 2013). Salburinal, a selective inhibitor of the phosphatase complexes that dephosphorylate p-eIF2α, elevates the level of p-eIF2α in ER-stressed mice (Boyce et al., 2005). Guanabenz selectively binds to GADD34 and inhibits the activity of GADD34/PP1 complex, attenuating p-eIF2α dephosphorylation during ER stress (Tsaytler et al., 2011). The PERK activator CCT020312 stimulates PERK-mediated eIF2α phosphorylation in the absence of ER stress (Stockwell et al., 2012). Interestingly, the therapeutic potential of these modulators of the PERK-eIF2α pathway has been tested in animal models of human diseases. Treatment with the PERK inhibitor GSK2606414 attenuates protein translation inhibition induced by PERK activation and abrogates neurodegeneration and clinical symptoms in a mouse model of prion disease (Moreno et al., 2013). Administration of the PERK inhibitor GSK2656157 inhibits multiple human tumor xenografts growth in mice by suppressing angiogenesis (Atkins et al., 2013). Salubrinal treatment reduces ER accumulation of α-synuclein in neurons and extends the life span of A53T α-synuclein transgenic mice, a mouse model of Parkinson's disease (Colla et al., 2012). Guanabenz treatment increases the level of p-eIF2α, reduces accumulation of mutant superoxide dismutase type 1 (mtSOD1), attenuates motor neuron death, and prolongs survival of mtSOD1 transgenic mice, a mouse model of amyotrophic lateral sclerosis (Wang et al., 2014).

Using genetic approaches, the studies described above well-documented the cytoprotective effects of the PERK-eIF2α pathway on both mature and remyelinating oligodendrocytes in mouse models of MS (Lin et al., 2013, 2014a; Hussien et al., 2014). Oligodendrocytes are not only highly sensitive to activation of the PERK-eIF2α pathway but also tolerate the activation extremely well (Lin and Popko, 2009; Lin et al., 2014b; Lin, 2015). Importantly, CHOP, a major pro-apoptotic transcription factor induced by PERK activation, has no significant effect on oligodendrocyte viability (Gow and Wrabetz, 2009; Lin et al., 2014b). These genetic studies indicate that the PERK-eIF2α pathway would be an ideal target to develop therapeutic strategies for oligodendrocyte protection in MS patients. Consistently, data from studies using small chemical compounds suggest the therapeutic potential of activation of the PERK-eIF2α pathway in models of MS. A report showed that Salburinal treatment elevates the level of p-eIF2α and attenuates IFN-γ-induced myelinating oligodendrocyte death and myelin loss in hippocampal slice cultures, an *in vitro* model of myelination (Lin et al., 2008). Moreover, a very recent report showed that Guanabenz treatment increases the level of p-eIF2α and protects myelinating oligodendrocytes against the detrimental effects of IFN-γ in cerebellar slice cultures and in transgenic mice that express IFN-γ in the CNS. This report also showed that Guanabenz treatment elevates the level of p-eIF2α, ameliorates EAE disease severity, and attenuates EAE-induced oligodendrocyte death and demyelination in the CNS (Way et al., 2015). Additionally, Guanabenz treatment does not alter T cell proliferation or cytokine production in the peripheral immune system, but suppresses CD4 T cell activation in the CNS during EAE. Although data derived from mouse models of MS are compelling, it is a formidable challenge to develop effective therapeutic strategies to activate the PERK-eIF2α pathway for MS treatment, without causing side effects.

## Future directions

The ER is essential for the biosynthesis of myelin lipids and proteins. The volume of the ER increases dramatically in the cytoplasm of oligodendrocytes during the active phase of myelination (Wollmann et al., 1981). However, the mechanisms that regulate the ER expansion during oligodendrocyte development remain unexplored. Recent studies suggest the UPR plays a role in the ER biogenesis (Brewer and Hendershot, 2005; Aragon et al., 2012). Activation of the IRE1-XBP1 pathway directs the expansion of the ER during the differentiation of B lymphocytes into antibody-secreting cells (Iwakoshi et al., 2003; Sriburi et al., 2004). Activation of ATF6 also has the ability to drive the ER expansion independent of sXBP1 (Bommiasamy et al., 2009). In contrast, the PERK-eIF2α pathway does not participate in ER expansion during the development of antibody-secreting cells (Gass et al., 2008; Aragon et al., 2012). Although activation of PERK and ATF6 has been observed in oligodendrocytes, deletion of either PERK or ATF6 has no discernible effect on oligodendrocyte development (Yamamoto et al., 2010; Hussien et al., 2014). Since the functions of the PERK pathway and the ATF6 pathway overlap considerably (Marciniak and Ron, 2006; Walter and Ron, 2011), the minimal effect of either PERK or ATF6 deletion on oligodendrocyte development might suggest that they compensate for each other. It would be interesting to determine the role of the UPR in the ER biogenesis during oligodendrocyte development using PERK and ATF6 double knock-out mice.

While the actions of PERK activation in oligodendrocytes in immune-mediated demyelinating diseases have been studied extensively, the underlying mechanisms through which PERK mediates protection remain unknown and deserve further investigation. Moreover, activation of the PERK pathway has been observed in other cell types in MS and EAE demyelinating lesions, including T cells, microglia/macrophages, and astrocytes (Chakrabarty et al., 2004; Mháille et al., 2008; Cunnea et al., 2011). A number of studies have shown that the PERK pathway regulates the functions of T cells, macrophages, and astrocytes (Woo et al., 2009; Wheatley et al., 2011; Meares et al., 2014). Therefore, it would be important to dissect the precise role of PERK signaling in these cell types in the pathogenesis of EAE using cell-specific PERK knock-out mice. Moreover, future studies will need to assess the role of the ATF6 pathway in oligodendrocytes and other cell types in models of MS.

For many years, the focus of MS research has been on T cell-mediated demyelination of the white matter. Recent studies, however, have shown that neurodegeneration is not only an early event but also the primary cause of chronic disability in MS (Dutta and Trapp, 2011; Popescu and Lucchinetti, 2012). It is well-documented that axon transection and loss occur early in the CNS of MS patients (Trapp and Nave, 2008). Significant neuron loss has also been observed in the CNS gray matter of MS patients, including the cerebral cortex, hippocampus, thalamus, and spinal cord (Papadopoulos et al., 2009; Vogt et al., 2009). Moreover, magnetic resonance image studies show that progressive brain atrophy in MS patients correlates well with disability (Vigeveno et al., 2012). Interestingly, several studies have shown that both axon degeneration and neuron loss occur early in the EAE disease course (Lassmann, 2010). Although the current predominant view is that inflammation is ultimately responsible for neurodegeneration in MS and EAE (Glass et al., 2010; Siffrin et al., 2010), the actual steps leading from the immune attack on oligodendrocytes and myelin to neurodegeneration remain elusive. A number of studies have shown that the UPR influences, positively or negatively, neuron viability in various neurodegenerating diseases, including Alzheimer's disease, Parkinson's disease, amyotrophic lateral sclerosis, and prion disease (Doyle et al., 2011; Hetz and Mollereau, 2014). Importantly, a report showed that the levels of p-eIF2α and CHOP are increased in neurons in the CNS of EAE mice as compared to naïve mice (Ní Fhlathartaigh et al., 2013). Thus, there is a possibility that the UPR regulates neuron viability in MS and EAE. It is therefore important to determine the role of the individual branches of the UPR in neurons during EAE using neuron-specific knock-out mice.

In summary, previous studies have demonstrated the cytoprotective effects of the PERK branch of the UPR on oligodendrocytes in models of MS. Future studies will need to assess the role of other branches of the UPR in oligodendrocytes and the roles of individual branches of the UPR in other cell types involved in MS, particularly neurons, in this disease. The knowledge gained from these studies would provide a foundation to develop therapeutic strategies that protects both oligodendrocytes and neurons in patients with MS.

### Conflict of interest statement

The authors declare that the research was conducted in the absence of any commercial or financial relationships that could be construed as a potential conflict of interest.

## References

[B1] Anitei M. Pfeiffer S. E. (2006). Myelin biogenesis: sorting out protein trafficking. Curr. Biol. 16, R418–R421. 10.1016/j.cub.2006.05.010 16753556

[B2] Aragon I. V. Barrington R. A. Jackowski S. Mori K. Brewer J. W. (2012). The specialized unfolded protein response of B lymphocytes: ATF6α-independent development of antibody-secreting B cells. Mol. Immunol. 51, 347–355. 10.1016/j.molimm.2012.04.001 22555069 PMC3358488

[B3] Atkins C. Liu Q. Minthorn E. Zhang S. Y. Figueroa D. J. Moss K.. (2013). Characterization of a novel PERK kinase inhibitor with antitumor and antiangiogenic activity. Cancer. Res. 73, 1993–2002. 10.1158/0008-5472.CAN-12-3109 23333938

[B4] Axten J. M. Medina J. R. Feng Y. Shu A. Romeril S. P. Grant S. W.. (2012). Discovery of 7-methyl-5-(1-{[3-(trifluoromethyl)phenyl]acetyl}-2,3-dihydro-1H-indol-5-yl)-7H-pyrrolo[2,3-d]pyrimidin-4-amine (GSK2606414), a potent and selective first-in-class inhibitor of protein kinase R (PKR)-like endoplasmic reticulum kinase (PERK). J. Med. Chem. 55, 7193–7207. 10.1021/jm300713s 22827572

[B5] Balabanov R. Strand K. Goswami R. McMahon E. Begolka W. Miller S. D.. (2007). Interferon-gamma-oligodendrocyte interactions in the regulation of experimental autoimmune encephalomyelitis. J. Neurosci. 27, 2013–2024. 10.1523/JNEUROSCI.4689-06.2007 17314297 PMC6673565

[B6] Bankston A. N. Mandler M. D. Feng Y. (2013). Oligodendroglia and neurotrophic factors in neurodegeneration. Neurosci. Bull. 29, 216–228. 10.1007/s12264-013-1321-3 23558590 PMC4020141

[B7] Barnett M. H. Prineas J. W. (2004). Relapsing and remitting multiple sclerosis: pathology of the newly forming lesion. Ann. Neurol. 55, 458–468. 10.1002/ana.20016 15048884

[B8] Baxter A. G. (2007). The origin and application of experimental autoimmune encephalomyelitis. Nat. Rev. Immunol. 7, 904–912. 10.1038/nri2190 17917672

[B9] Bommiasamy H. Back S. H. Fagone P. Lee K. Meshinchi S. Vink E.. (2009). ATF6alpha induces XBP1-independent expansion of the endoplasmic reticulum. J. Cell. Sci. 122, 1626–1636. 10.1242/jcs.045625 19420237 PMC2680102

[B10] Boyce M. Bryant K. F. Jousse C. Long K. Harding H. P. Scheuner D.. (2005). A selective inhibitor of eIF2alpha dephosphorylation protects cells from ER stress. Science 307, 935–939. 10.1126/science.1101902 15705855

[B11] Bradl M. Lassmann H. (2010). Oligodendrocytes: biology and pathology. Acta Neuropathol. 119, 37–53. 10.1007/s00401-009-0601-5 19847447 PMC2799635

[B12] Brewer J. W. Hendershot L. M. (2005). Building an antibody factory: a job for the unfolded protein response. Nat. Immunol. 6, 23–29. 10.1038/ni1149 15611778

[B13] Cao S. S. Kaufman R. J. (2014). Endoplasmic reticulum stress and oxidative stress in cell fate decision and human disease. Antioxid. Redox Signal. 21, 396–413. 10.1089/ars.2014.5851 24702237 PMC4076992

[B14] Chakrabarty A. Danley M. M. LeVine S. M. (2004). Immunohistochemical localization of phosphorylated protein kinase R and phosphorylated eukaryotic initiation factor-2 alpha in the central nervous system of SJL mice with experimental allergic encephalomyelitis. J. Neurosci. Res. 76, 822–833. 10.1002/jnr.20125 15160394

[B15] Chakrabarty A. Fleming K. K. Marquis J. G. LeVine S. M. (2005). Quantifying immunohistochemical staining of phospho-eIF2alpha, heme oxygenase-2 and NADPH cytochrome P450 reductase in oligodendrocytes during experimental autoimmune encephalomyelitis. J. Neurosci. Methods 144, 227–234. 10.1016/j.jneumeth.2004.11.010 15910982

[B16] Chen Y. Brandizzi F. (2013). IRE1: ER stress sensor and cell fate executor. Trends Cell Biol. 23, 547–555. 10.1016/j.tcb.2013.06.005 23880584 PMC3818365

[B17] Colla E. Jensen P. H. Pletnikova O. Troncoso J. C. Glabe C. Lee M. K. (2012). Accumulation of toxic α-synuclein oligomer within endoplasmic reticulum occurs in α-synucleinopathy *in vivo*. J. Neurosci. 32, 3301–3305. 10.1523/JNEUROSCI.5368-11.2012 22399752 PMC3548448

[B18] Corbin J. G. Kelly D. Rath E. M. Baerwald K. D. Suzuki K. Popko B. (1996). Targeted CNS expression of interferon-gamma in transgenic mice leads to hypomyelination, reactive gliosis, and abnormal cerebellar development. Mol. Cell Neurosci. 7, 354–370. 10.1006/mcne.1996.0026 8812062

[B19] Cox D. J. Strudwick N. Ali A. A. Paton A. W. Paton J. C. Schröder M. (2011). Measuring signaling by the unfolded protein response. Methods Enzymol. 491, 261–292. 10.1016/B978-0-12-385928-0.00015-8 21329805

[B20] Csala M. Bánhegyi G. Benedetti A. (2006). Endoplasmic reticulum: a metabolic compartment. FEBS Lett. 580, 2160–2165. 10.1016/j.febslet.2006.03.050 16580671

[B21] Cunnea P. Mháille A. N. McQuaid S. Farrell M. McMahon J. FitzGerald U. (2011). Expression profiles of endoplasmic reticulum stress-related molecules in demyelinating lesions and multiple sclerosis. Mult. Scler. 17, 808–818. 10.1177/1352458511399114 21382862

[B22] Cwiklinska H. Mycko M. P. Luvsannorov O. Walkowiak B. Brosnan C. F. Raine C. S.. (2003). Heat shock protein 70 associations with myelin basic protein and proteolipid protein in multiple sclerosis brains. Int. Immunol. 15, 241–249. 10.1093/intimm/dxg022 12578854

[B23] D'Antonio M. Feltri M. L. Wrabetz L. (2009). Myelin under stress. J. Neurosci. Res. 87, 3241–3249. 10.1002/jnr.22066 19330777 PMC3076940

[B24] Deng J. Lu P. D. Zhang Y. Scheuner D. Kaufman R. J. Sonenberg N.. (2004). Translational repression mediates activation of nuclear factor kappa B by phosphorylated translation initiation factor 2. Mol. Cell. Biol. 24, 10161–10168. 10.1128/MCB.24.23.10161-10168.2004 15542827 PMC529034

[B25] Denic A. Johnson A. J. Bieber A. J. Warrington A. E. Rodriguez M. Pirko I. (2011). The relevance of animal models in multiple sclerosis research. Pathophysiology 18, 21–29. 10.1016/j.pathophys.2010.04.004 20537877 PMC3858209

[B26] Deslauriers A. M. Afkhami-Goli A. Paul A. M. Bhat R. K. Acharjee S. Ellestad K. K.. (2011). Neuroinflammation and endoplasmic reticulum stress are coregulated by crocin to prevent demyelination and neurodegeneration. J. Immunol. 187, 4788–4799. 10.4049/jimmunol.1004111 21964030

[B27] Doyle K. M. Kennedy D. Gorman A. M. Gupta S. Healy S. J. Samali A. (2011). Unfolded proteins and endoplasmic reticulum stress in neurodegenerative disorders. J. Cell. Mol. Med. 15, 2025–2039. 10.1111/j.1582-4934.2011.01374.x 21722302 PMC4394214

[B28] Dutta R. Trapp B. D. (2011). Mechanisms of neuronal dysfunction and degeneration in multiple sclerosis. Prog. Neurobiol. 93, 1–12. 10.1016/j.pneurobio.2010.09.005 20946934 PMC3030928

[B29] Fancy S. P. Chan J. R. Baranzini S. E. Franklin R. J. Rowitch D. H. (2011). Myelin regeneration: a recapitulation of development? Annu. Rev. Neurosci. 34, 21–43. 10.1146/annurev-neuro-061010-113629 21692657

[B30] Ferber I. A. Brocke S. Taylor-Edwards C. Ridgway W. Dinisco C. Steinman L.. (1996). Mice with a disrupted IFN-gamma gene are susceptible to the induction of experimental autoimmune encephalomyelitis (EAE). J. Immunol. 156, 5–7. 8598493

[B31] Franklin R. J. (2002). Why does remyelination fail in multiple sclerosis? Nat. Rev. Neurosci. 3, 705–714. 10.1038/nrn917 12209119

[B32] Franklin R. J. Ffrench-Constant C. (2008). Remyelination in the CNS: from biology to therapy. Nat. Rev. Neurosci. 9, 839–855. 10.1038/nrn2480 18931697

[B33] Frohman E. M. Racke M. K. Raine C. S. (2006). Multiple sclerosis–the plaque and its pathogenesis. N. Engl. J. Med. 354, 942–955. 10.1056/NEJMra052130 16510748

[B34] Gade P. Ramachandran G. Maachani U. B. Rizzo M. A. Okada T. Prywes R.. (2012). An IFN-γ-stimulated ATF6-C/EBP-β-signaling pathway critical for the expression of Death Associated Protein Kinase 1 and induction of autophagy. Proc. Natl. Acad. Sci. U.S.A. 109, 10316–10321. 10.1073/pnas.1119273109 22699507 PMC3387052

[B35] Gass J. N. Jiang H. Y. Wek R. C. Brewer J. W. (2008). The unfolded protein response of B-lymphocytes: PERK-independent development of antibody-secreting cells. Mol. Immunol. 45, 1035–1043. 10.1016/j.molimm.2007.07.029 17822768 PMC2677759

[B36] Glass C. K. Saijo K. Winner B. Marchetto M. C. Gage F. H. (2010). Mechanisms underlying inflammation in neurodegeneration. Cell 140, 918–934. 10.1016/j.cell.2010.02.016 20303880 PMC2873093

[B37] Glembotski C. C. (2014). Roles for ATF6 and the sarco/endoplasmic reticulum protein quality control system in the heart. J. Mol. Cell Cardiol. 71, 11–15. 10.1016/j.yjmcc.2013.09.018 24140798 PMC4157898

[B38] Gold R. Linington C. Lassmann H. (2006). Understanding pathogenesis and therapy of multiple sclerosis via animal models: 70 years of merits and culprits in experimental autoimmune encephalomyelitis research. Brain 129, 1953–1971. 10.1093/brain/awl075 16632554

[B39] Goverman J. (2009). Autoimmune T cell responses in the central nervous system. Nat. Rev. Immunol. 9, 393–407. 10.1038/nri2550 19444307 PMC2813731

[B40] Gow A. Wrabetz L. (2009). CHOP and the endoplasmic reticulum stress response in myelinating glia. Curr. Opin. Neurobiol. 19, 505–510. 10.1016/j.conb.2009.08.007 19744850 PMC2787654

[B41] Harding H. P. Calfon M. Urano F. Novoa I. Ron D. (2002). Transcriptional and translational control in the mammalian unfolded protein response. Annu. Rev. Cell. Dev. Biol. 18, 575–599. 10.1146/annurev.cellbio.18.011402.160624 12142265

[B42] Harding H. P. Novoa I. Bertolotti A. Zeng H. Zhang Y. Urano F.. (2001). Translational regulation in the cellular response to biosynthetic load on the endoplasmic reticulum. Cold Spring Harb. Symp. Quant. Biol. 66, 499–508. 10.1101/sqb.2001.66.499 12762052

[B43] Harding H. P. Zhang Y. Ron D. (1999). Protein translation and folding are coupled by an endoplasmic-reticulum-resident kinase. Nature 397, 271–274. 10.1038/16729 9930704

[B44] Harding H. P. Zhang Y. Zeng H. Novoa I. Lu P. D. Calfon M.. (2003). An integrated stress response regulates amino acid metabolism and resistance to oxidative stress. Mol. Cell. 11, 619–633. 10.1016/S1097-2765(03)00105-9 12667446

[B45] Harding H. P. Zyryanova A. F. Ron D. (2012). Uncoupling proteostasis and development *in vitro* with a small molecule inhibitor of the pancreatic endoplasmic reticulum kinase, PERK. J. Biol. Chem. 287, 44338–44344. 10.1074/jbc.M112.428987 23148209 PMC3531748

[B46] Hetz C. (2012). The unfolded protein response: controlling cell fate decisions under ER stress and beyond. Nat. Rev. Mol. Cell. Biol. 13, 89–102. 10.1038/nrm3270 22251901

[B47] Hetz C. Chevet E. Harding H. P. (2013). Targeting the unfolded protein response in disease. Nat. Rev. Drug Discov. 12, 703–719. 10.1038/nrd3976 23989796

[B48] Hetz C. Mollereau B. (2014). Disturbance of endoplasmic reticulum proteostasis in neurodegenerative diseases. Nat. Rev. Neurosci. 15, 233–249. 10.1038/nrn3689 24619348

[B49] Hisahara S. Araki T. Sugiyama F. Yagami K. Suzuki M. Abe K.. (2000). Targeted expression of baculovirus p35 caspase inhibitor in oligodendrocytes protects mice against autoimmune-mediated demyelination. EMBO J. 19, 341–348. 10.1093/emboj/19.3.341 10654933 PMC305571

[B50] Hisahara S. Okano H. Miura M. (2003). Caspase-mediated oligodendrocyte cell death in the pathogenesis of autoimmune demyelination. Neurosci. Res. 46, 387–397. 10.1016/S0168-0102(03)00127-5 12871760

[B51] Hövelmeyer N. Hao Z. Kranidioti K. Kassiotis G. Buch T. Frommer F.. (2005). Apoptosis of Oligodendrocytes via Fas and TNF-R1 Is a Key Event in the Induction of Experimental Autoimmune Encephalomyelitis. J. Immunol. 175, 5875–5884. 10.4049/jimmunol.175.9.5875 16237080

[B52] Hu P. Han Z. Couvillon A. D. Kaufman R. J. Exton J. H. (2006). Autocrine tumor necrosis factor alpha links endoplasmic reticulum stress to the membrane death receptor pathway through IRE1alpha-mediated NF-kappaB activation and down-regulation of TRAF2 expression. Mol. Cell. Biol. 26, 3071–3084. 10.1128/MCB.26.8.3071-3084.2006 16581782 PMC1446932

[B53] Hussien Y. Cavener D. R. Popko B. (2014). Genetic inactivation of PERK signaling in mouse oligodendrocytes: normal developmental myelination with increased susceptibility to inflammatory demyelination. Glia 62, 680–691. 10.1002/glia.22634 24481666 PMC6342275

[B54] Iwakoshi N. N. Lee A. H. Vallabhajosyula P. Otipoby K. L. Rajewsky K. Glimcher L. H. (2003). Plasma cell differentiation and the unfolded protein response intersect at the transcription factor XBP-1. Nat. Immunol. 4, 321–329. 10.1038/ni907 12612580

[B55] Jiang H. Y. Wek S. A. McGrath B. C. Scheuner D. Kaufman R. J. Cavener D. R.. (2003). Phosphorylation of the alpha subunit of eukaryotic initiation factor 2 is required for activation of NF-kappaB in response to diverse cellular stresses. Mol. Cell. Biol. 23, 5651–5663. 10.1128/MCB.23.16.5651-5663.2003 12897138 PMC166326

[B56] Karin M. Lin A. (2002). NF-kappaB at the crossroads of life and death. Nat. Immunol. 3, 221–227. 10.1038/ni0302-221 11875461

[B57] Kaufman R. J. (1999). Stress signaling from the lumen of the endoplasmic reticulum: coordination of gene transcriptional and translational controls. Genes Dev. 13, 1211–1233. 10.1101/gad.13.10.1211 10346810

[B58] Kitamura M. (2011). Control of NF-κB and inflammation by the unfolded protein response. Int. Rev. Immunol. 30, 4–15. 10.3109/08830185.2010.522281 21235322

[B59] LaFerla F. M. Sugarman M. C. Lane T. E. Leissring M. A. (2000). Regional hypomyelination and dysplasia in transgenic mice with astrocyte-directed expression of interferon-gamma. J. Mol. Neurosci. 15, 45–59. 10.1385/JMN:15:1:45 11211236

[B60] Lassmann H. (2010). Axonal and neuronal pathology in multiple sclerosis: what have we learnt from animal models. Exp. Neurol. 225, 2–8. 10.1016/j.expneurol.2009.10.009 19840788

[B61] Lees J. R. Cross A. H. (2007). A little stress is good: IFN-gamma, demyelination, and multiple sclerosis. J. Clin. Invest. 117, 297–299. 10.1172/JCI31254 17273549 PMC1783822

[B62] Lin W. (2015). Impaired eIF2B activity in oligodendrocytes contributes to VWMD pathogenesis. Neural. Regen. Res. 10, 195–197 10.4103/1673-5374.152366 25883611 PMC4392660

[B63] Lin W. Bailey S. L. Ho H. Harding H. P. Ron D. Miller S. D.. (2007). The integrated stress response prevents demyelination by protecting oligodendrocytes against immune-mediated damage. J. Clin. Invest. 117, 448–456. 10.1172/JCI29571 17273557 PMC1783809

[B64] Lin W. Harding H. P. Ron D. Popko B. (2005). Endoplasmic reticulum stress modulates the response of myelinating oligodendrocytes to the immune cytokine interferon-gamma. J. Cell. Biol. 169, 603–612. 10.1083/jcb.200502086 15911877 PMC2171696

[B65] Lin W. Kemper A. Dupree J. L. Harding H. P. Ron D. Popko B. (2006). Interferon-gamma inhibits central nervous system remyelination through a process modulated by endoplasmic reticulum stress. Brain 129, 1306–1318. 10.1093/brain/awl044 16504972

[B66] Lin W. Kemper A. McCarthy K. D. Pytel P. Wang J. P. Campbell I. L.. (2004). Interferon-gamma induced medulloblastoma in the developing cerebellum. J. Neurosci. 24, 10074–10083. 10.1523/JNEUROSCI.2604-04.2004 15537876 PMC6730177

[B67] Lin W. Kunkler P. E. Harding H. P. Ron D. Kraig R. P. Popko B. (2008). Enhanced integrated stress response promotes myelinating oligodendrocyte survival in response to interferon-gamma. Am. J. Pathol. 173, 1508–1517. 10.2353/ajpath.2008.080449 18818381 PMC2570140

[B68] Lin W. Lin Y. (2010). Interferon-γ inhibits central nervous system myelination through both STAT1-dependent and STAT1-independent pathways. J. Neurosci. Res. 88, 2569–2577. 10.1002/jnr.22425 20648647 PMC2911948

[B69] Lin W. Lin Y. Li J. Fenstermaker A. G. Way S. W. Clayton B.. (2013). Oligodendrocyte-specific activation of PERK signaling protects mice against experimental autoimmune encephalomyelitis. J. Neurosci. 33, 5980–5991. 10.1523/JNEUROSCI.1636-12.2013 23554479 PMC3654380

[B70] Lin W. Popko B. (2009). Endoplasmic reticulum stress in disorders of myelinating cells. Nat. Neurosci. 12, 379–385. 10.1038/nn.2273 19287390 PMC2697061

[B71] Lin Y. Huang G. Jamison S. Li J. Harding H. P. Ron D.. (2014a). PERK activation preserves the viability and function of remyelinating oligodendrocytes in immune-mediated demyelinating diseases. Am. J. Pathol. 184, 507–519. 10.1016/j.ajpath.2013.10.009 24269558 PMC3906495

[B72] Lin Y. Jamison S. Lin W. (2012). Interferon-γ activates nuclear factor-κ B in oligodendrocytes through a process mediated by the unfolded protein response. PLoS ONE 7:e36408. 10.1371/journal.pone.0036408 22574154 PMC3344863

[B73] Lin Y. Pang X. Huang G. Jamison S. Fang J. Harding H. P.. (2014b). Impaired eukaryotic translation initiation factor 2B activity specifically in oligodendrocytes reproduces the pathology of vanishing white matter disease in mice. J. Neurosci. 34, 12182–12191. 10.1523/JNEUROSCI.1373-14.2014 25186761 PMC4152613

[B74] Lu P. D. Jousse C. Marciniak S. J. Zhang Y. Novoa I. Scheuner D.. (2004). Cytoprotection by pre-emptive conditional phosphorylation of translation initiation factor 2. EMBO J. 23, 169–179. 10.1038/sj.emboj.7600030 14713949 PMC1271668

[B75] Marciniak S. J. Ron D. (2006). Endoplasmic reticulum stress signaling in disease. Physiol. Rev. 86, 1133–1149. 10.1152/physrev.00015.2006 17015486

[B76] Marciniak S. J. Yun C. Y. Oyadomari S. Novoa I. Zhang Y. Jungreis R.. (2004). CHOP induces death by promoting protein synthesis and oxidation in the stressed endoplasmic reticulum. Genes Dev. 18, 3066–3077. 10.1101/gad.1250704 15601821 PMC535917

[B77] Matsushima G. K. Morell P. (2001). The neurotoxicant, cuprizone, as a model to study demyelination and remyelination in the central nervous system. Brain Pathol. 11, 107–116. 10.1111/j.1750-3639.2001.tb00385.x 11145196 PMC8098267

[B78] Maurel M. Chevet E. Tavernier J. Gerlo S. (2014). Getting RIDD of RNA: IRE1 in cell fate regulation. Trends Biochem. Sci. 39, 245–554. 10.1016/j.tibs.2014.02.008 24657016

[B79] McGuire C. Prinz M. Beyaert R. van Loo G. (2013). Nuclear factor kappa B (NF-κB) in multiple sclerosis pathology. Trends Mol. Med. 19, 604–613. 10.1016/j.molmed.2013.08.001 24007818

[B80] McGuire C. Volckaert T. Wolke U. Sze M. de Rycke R. Waisman A.. (2010). Oligodendrocyte-specific FADD deletion protects mice from autoimmune-mediated demyelination. J. Immunol. 185, 7646–7653. 10.4049/jimmunol.1000930 21068410

[B81] McMahon J. M. McQuaid S. Reynolds R. FitzGerald U. F. (2012). Increased expression of ER stress- and hypoxia-associated molecules in grey matter lesions in multiple sclerosis. Mult. Scler. 18, 1437–1447. 10.1177/1352458512438455 22354737

[B82] Meares G. P. Liu Y. Rajbhandari R. Qin H. Nozell S. E. Mobley J. A.. (2014). PERK-dependent activation of JAK1 and STAT3 contributes to endoplasmic reticulum stress-induced inflammation. Mol. Cell Biol. 34, 3911–3925. 10.1128/MCB.00980-14 25113558 PMC4187715

[B83] Merrill J. E. Kono D. H. Clayton J. Ando D. G. Hinton D. R. Hofman F. M. (1992). Inflammatory leukocytes and cytokines in the peptide-induced disease of experimental allergic encephalomyelitis in SJL and B10.PL mice. *Proc. Natl. Aacd. Sci*. U.S.A. 89, 574–578. 10.1073/pnas.89.2.574 1370583 PMC48281

[B84] Mháille A. N. McQuaid S. Windebank A. Cunnea P. McMahon J. Samali A.. (2008). Increased expression of endoplasmic reticulum stress-related signaling pathway molecules in multiple sclerosis lesions. J. Neuropathol. Exp. Neurol. 67, 200–211. 10.1097/NEN.0b013e318165b239 18344911

[B85] Mincheva-Tasheva S. Soler R. M. (2013). NF-κB signaling pathways: role in nervous system physiology and pathology. Neuroscientist 19, 175–194. 10.1177/1073858412444007 22785105

[B86] Mor F. Cohen I. R. (1992). T cells in the lesion of experimental autoimmune encephalomyelitis. Enrichment for reactivities to myelin basic protein and to heat shock proteins. J. Clin. Invest. 90, 2447–2455. 10.1172/JCI116136 1281835 PMC443401

[B87] Moreno J. A. Halliday M. Molloy C. Radford H. Verity N. Axten J. M.. (2013). Oral treatment targeting the unfolded protein response prevents neurodegeneration and clinical disease in prion-infected mice. Sci. Transl. Med. 5, 206ra138. 10.1126/scitranslmed.3006767 24107777

[B88] Mühl H. Pfeilschifter J. (2003). Anti-inflammatory properties of pro-inflammatory interferon-gamma. Int. Immunopharmacol. 3, 1247–1255. 10.1016/S1567-5769(03)00131-0 12890422

[B89] Mycko M. P. Papoian R. Boschert U. Raine C. S. Selmaj K. W. (2004). Microarray gene expression profiling of chronic active and inactive lesions in multiple sclerosis. Clin. Neurol. Neurosurg. 106, 223–229. 10.1016/j.clineuro.2004.02.019 15177772

[B90] Nave K. A. Werner H. B. (2014). Myelination of the nervous system: mechanisms and functions. Annu. Rev. Cell Dev. Biol. 30, 503–533. 10.1146/annurev-cellbio-100913-013101 25288117

[B91] Ní Fhlathartaigh M. McMahon J. Reynolds R. Connolly D. Higgins E. Fitzgerald U.. (2013). Calreticulin and other components of endoplasmic reticulum stress in rat and human inflammatory demyelination. Acta Neuropathol. Commun. 1:37. 10.1186/2051-5960-1-37 24252779 PMC3893522

[B92] Novoa I. Zeng H. Harding H. P. Ron D. (2001). Feedback inhibition of the unfolded protein response by GADD34-mediated dephosphorylation of eIF2alpha. J. Cell. Biol. 153, 1011–1022. 10.1083/jcb.153.5.1011 11381086 PMC2174339

[B93] Ohri S. S. Hetman M. Whittemore S. R. (2013). Restoring endoplasmic reticulum homeostasis improves functional recovery after spinal cord injury. Neurobiol. Dis. 58, 29–37. 10.1016/j.nbd.2013.04.021 23659896 PMC3748169

[B94] Panitch H. S. Hirsch R. L. Schindler J. Johnson K. P. (1987). Treatment of multiple sclerosis with gamma interferon: exacerbations associated with activation of the immune system. Neurology 37, 1097–1102. 10.1212/WNL.37.7.1097 3110648

[B95] Papadopoulos D. Dukes S. Patel R. Nicholas R. Vora A. Reynolds R. (2009). Substantial archaeocortical atrophy and neuronal loss in multiple sclerosis. Brain Pathol. 19, 238–253. 10.1111/j.1750-3639.2008.00177.x 18492094 PMC8094861

[B96] Pavitt G. D. Proud C. G. (2009). Protein synthesis and its control in neuronal cells with a focus on vanishing white matter disease. Biochem. Soc. Trans. 37, 1298–1310. 10.1042/BST0371298 19909266

[B97] Pfeiffer S. E. Warrington A. E. Bansal R. (1993). The oligodendrocyte and its many cellular processes. Trends Cell. Biol. 3, 191–197. 10.1016/0962-8924(93)90213-K 14731493

[B98] Popescu B. F. Lucchinetti C. F. (2012). Pathology of demyelinating diseases. Annu. Rev. Pathol. 7, 185–217. 10.1146/annurev-pathol-011811-132443 22313379

[B99] Popko B. Baerwald K. D. (1999). Oligodendroglial response to the immune cytokine interferon gamma. Neurochem. Res. 24, 331–338. 10.1023/A:1022586726510 9972883

[B100] Popko B. Corbin J. G. Baerwald K. D. Dupree J. Garcia A. M. (1997). The effects of interferon-gamma on the central nervous system. Mol. Neurobiol. 14, 19–35. 10.1007/BF02740619 9170099 PMC7091409

[B101] Prineas J. W. Parratt J. D. (2012). Oligodendrocytes and the early multiple sclerosis lesion. Ann. Neurol. 72, 18–31. 10.1002/ana.23634 22829266

[B102] Qi L. Yang L. Chen H. (2011). Detecting and quantitating physiological endoplasmic reticulum stress. Methods Enzymol. 490, 137–146. 10.1016/B978-0-12-385114-7.00008-8 21266248 PMC3374842

[B103] Ramana C. V. Gil M. P. Schreiber R. D. Stark G. R. (2002). Stat1-dependent and -independent pathways in IFN-gamma-dependent signaling. Trends Immunol. 23, 96–101. 10.1016/S1471-4906(01)02118-4 11929133

[B104] Ren Z. Wang Y. Tao D. Liebenson D. Liggett T. Goswami R.. (2011). Overexpression of the dominant-negative form of interferon regulatory factor 1 in oligodendrocytes protects against experimental autoimmune encephalomyelitis. J. Neurosci. 31, 8329–8341. 10.1523/JNEUROSCI.1028-11.2011 21653838 PMC3770885

[B105] Scheper G. C. Proud C. G. van der Knaap M. S. (2006). Defective translation initiation causes vanishing of cerebral white matter. Trends Mol. Med. 12, 159–166. 10.1016/j.molmed.2006.02.006 16545608

[B106] Schröder M. (2008). Endoplasmic reticulum stress responses. Cell Mol. Life Sci. 65, 862–894. 10.1007/s00018-007-7383-5 18038217 PMC11131897

[B107] Shen J. Chen X. Hendershot L. Prywes R. (2002). ER stress regulation of ATF6 localization by dissociation of BiP/GRP78 binding and unmasking of Golgi localization signals. Dev. Cell 3, 99–111. 10.1016/S1534-5807(02)00203-4 12110171

[B108] Siffrin V. Vogt J. Radbruch H. Nitsch R. Zipp F. (2010). Multiple sclerosis - candidate mechanisms underlying CNS atrophy. Trends Neurosci. 33, 202–210. 10.1016/j.tins.2010.01.002 20153532

[B109] Southwood C. M. Garbern J. Jiang W. Gow A. (2002). The unfolded protein response modulates disease severity in Pelizaeus-Merzbacher disease. Neuron 36, 585–596. 10.1016/S0896-6273(02)01045-0 12441049 PMC4603660

[B110] Sriburi R. Jackowski S. Mori K. Brewer J. W. (2004). XBP1: a link between the unfolded protein response, lipid biosynthesis, and biogenesis of the endoplasmic reticulum. J. Cell. Biol. 167, 35–41. 10.1083/jcb.200406136 15466483 PMC2172532

[B111] Steinman L. (1999). Assessment of animal models for MS and demyelinating disease in the design of rational therapy. Neuron 24, 511–514. 10.1016/S0896-6273(00)81107-1 10595504

[B112] Stidworthy M. F. Genoud S. Suter U. Mantei N. Franklin R. J. (2003). Quantifying the early stages of remyelination following cuprizone-induced demyelination. Brain Pathol. 13, 329–339. 10.1111/j.1750-3639.2003.tb00032.x 12946022 PMC8095752

[B113] Stockwell S. R. Platt G. Barrie S. E. Zoumpoulidou G. Te Poele R. H. Aherne G. W.. (2012). Mechanism-based screen for G1/S checkpoint activators identifies a selective activator of EIF2AK3/PERK signalling. PLoS ONE 7:e28568. 10.1371/journal.pone.0028568 22253692 PMC3257223

[B114] Tabas I. Ron D. (2011). Integrating the mechanisms of apoptosis induced by endoplasmic reticulum stress. Nat. Cell. Biol. 13, 184–190. 10.1038/ncb0311-184 21364565 PMC3107571

[B115] Trapp B. D. Nave K. A. (2008). Multiple sclerosis: an immune or neurodegenerative disorder? Annu. Rev. Neurosci. 31, 247–269. 10.1146/annurev.neuro.30.051606.094313 18558855

[B116] Tsaytler P. Harding H. P. Ron D. Bertolotti A. (2011). Selective inhibition of a regulatory subunit of protein phosphatase 1 restores proteostasis. Science 332, 91–94. 10.1126/science.1201396 21385720

[B117] van der Star B. J. Vogel D. Y. Kipp M. Puentes F. Baker D. Amor S. (2012). *In vitro* and *in vivo* models of multiple sclerosis. CNS Neurol. Disord. Drug Targets 11, 570–588. 10.2174/187152712801661284 22583443

[B118] Vigeveno R. M. Wiebenga O. T. Wattjes M. P. Geurts J. J. Barkhof F. (2012). Shifting imaging targets in multiple sclerosis: from inflammation to neurodegeneration. J. Magn. Reson. Imaging. 36, 1–19. 10.1002/jmri.23578 22696123

[B119] Vogt J. Paul F. Aktas O. Müller-Wielsch K. Dörr J. Dörr S.. (2009). Lower motor neuron loss in multiple sclerosis and experimental autoimmune encephalomyelitis. Ann. Neurol. 66, 310–322. 10.1002/ana.21719 19798635

[B120] Walter P. Ron D. (2011). The unfolded protein response: from stress pathway to homeostatic regulation. Science 334, 1081–1086. 10.1126/science.1209038 22116877

[B121] Wang L. Popko B. Tixier E. Roos R. P. (2014). Guanabenz, which enhances the unfolded protein response, ameliorates mutant SOD1-induced amyotrophic lateral sclerosis. Neurobiol. Dis. 71, 317–324. 10.1016/j.nbd.2014.08.010 25134731 PMC4179984

[B122] Wang M. Kaufman R. J. (2014). The impact of the endoplasmic reticulum protein-folding environment on cancer development. Nat. Rev. Cancer 14, 581–597. 10.1038/nrc3800 25145482

[B123] Wang S. Kaufman R. J. (2012). The impact of the unfolded protein response on human disease. J. Cell. Biol. 197, 857–867. 10.1083/jcb.201110131 22733998 PMC3384412

[B124] Way S. W. Podojil J. R. Clayton B. L. Zaremba A. Collins T. L. Kunjamma R. B.. (2015). Pharmaceutical integrated stress response enhancement protects oligodendrocytes and provides a potential multiple sclerosis therapeutic. Nat. Commun. 6, 6532. 10.1038/ncomms7532 25766071 PMC4360920

[B125] Wheatley A. K. Kramski M. Alexander M. R. Toe J. G. Center R. J. Purcell D. F. (2011). Co-expression of miRNA targeting the expression of PERK, but not PKR, enhances cellular immunity from an HIV-1 Env DNA vaccine. PLoS ONE 6:e18225. 10.1371/journal.pone.0018225 21464971 PMC3064671

[B126] Wheeler R. D. Owens T. (2005). The changing face of cytokines in the brain: perspectives from EAE. Curr. Pharm. Des. 11, 1031–1037. 10.2174/1381612053381657 15777252

[B127] Willenborg D. O. Fordham S. Bernard C. C. Cowden W. B. Ramshaw I. A. (1996). IFN-gamma plays a critical down-regulatory role in the induction and effector phase of myelin oligodendrocyte glycoprotein-induced autoimmune encephalomyelitis. J. Immunol. 157, 3223–3227. 8871615

[B128] Wollmann R. L. Szuchet S. Barlow J. Jerkovic M. (1981). Ultrastructural changes accompanying the growth of isolated oligodendrocytes. J. Neurosci. Res. 6, 757–769. 10.1002/jnr.490060610 7334534

[B129] Woo C. W. Cui D. Arellano J. Dorweiler B. Harding H. Fitzgerald K. A.. (2009). Adaptive suppression of the ATF4-CHOP branch of the unfolded protein response by toll-like receptor signalling. Nat. Cell. Biol. 11, 1473–1480. 10.1038/ncb1996 19855386 PMC2787632

[B130] Yamamoto K. Takahara K. Oyadomari S. Okada T. Sato T. Harada A.. (2010). Induction of liver steatosis and lipid droplet formation in ATF6alpha-knockout mice burdened with pharmacological endoplasmic reticulum stress. Mol. Biol. Cell. 21, 2975–2986. 10.1091/mbc.E09-02-0133 20631254 PMC2929991

[B131] Yan J. Greer J. M. (2008). NF-kappa B, a potential therapeutic target for the treatment of multiple sclerosis. CNS Neurol. Disord. Drug Targets 7, 536–557. 10.2174/187152708787122941 19128210

[B132] Zhang K. Kaufman R. J. (2008). From endoplasmic-reticulum stress to the inflammatory response. Nature 454, 455–462. 10.1038/nature07203 18650916 PMC2727659

